# Two Interesting Cases

**Published:** 1893-04

**Authors:** B. W. Mason

**Affiliations:** Sheridan, Ark.


### *CORRESPONDENCE.*

---

### TWO INTERESTING CASES.

---

SHERIDAN, ARK., February 13, 1893.

On the 22d of January last I was called to see a lady, white, forty years old ; had some fever, complained of pain in stomach and soreness under scapula. I diagnosed remittent malarial fever and put her upon quinine and told her husband to report in a few days if she was no better. Five days later he reported that for two or three days after my visit she seemed better, but wanted me to go to see her, as the pain in the stomach "was worse." I found her with slight fever and the pain in the stomach severe with cramping. I gave her calomel and put her upon subnit. bismuth. Called early next morning ; found her apparently better. The calomel had acted ; one large worm was discharged. I was sent for hurriedly next morning ; found her suffering severe pains in her stomach, with cramping. This continued until night, when she vomited four large worms. I put her upon santonin and purged her with castor oil. Upon this treatment she has passed four large worms by the bowels, and has vomited thirty-two, the smallest being seven inches long.

She has been easy now five days and is improving. This case was of interest to me, for in a practice of twenty-two years this is the first case of the kind I have ever seen, and I was surprised that so few of the worms were gotten rid of through the bowels.

I had a case of labor some time ago that was being attended by a "female," who goes to women "just for accommodation." The patient was well-nigh exhausted. Upon an examination, I found the rim of the os uteri was for the sixteenth or eighth of an inch deep of a hard, almost cartilaginous substance, which prevented dilatation. With blunt scissors I cut through this substance posteriorly, anteriorly and laterally, when she was delivered of a fœtus weighing seven or eight pounds. She made a good recovery. I was in hopes that during the "lying-in" the hard rim would disappear, but it did not. I was told that her sister had died some few years ago in labor and was not delivered.

Respectfully,

B. W. MASON.

---

### THE "HISTORY" IN SUSPECTED SYPHILIS.

Dr. Jas. C. McGuire, of Washington, in an article on the diagnosis of skin diseases, Virginia *Medical Monthly*, February, 1893, mentions case with papular eruption, and a mucous patch on lip. Diagnosed syphilis. Patient said skin *itched* very much; rheumatic pains during *day* only; no memory of sore throat; a widow six years; altogether a non-syphilitic history. The doctor learned of a syphilitic retinitis; syphilitic remedies removed eruption.

---

### PEDICULI PUBIS.

Dr. Nedzweidcki (*Med. Neuigkeiten*) regards benzine as the most efficient remedy in the treatment of pediculi pubis. It is used just as it appears in commerce, and thus forms the cleanest, most efficient and handiest remedy for the destruction of pediculi pubis and capitis. The affected parts must be bathed in the fluid for three to five minutes. The parasites and their eggs are killed at once. Generally one application is sufficient, even in the most difficult cases. The presence of eczematous spots does not contraindicate its use.—*Lancet-Clinic.*

